# Identifying Candidate Flavonoids for Non-Alcoholic Fatty Liver Disease by Network-Based Strategy

**DOI:** 10.3389/fphar.2022.892559

**Published:** 2022-05-26

**Authors:** Won-Yung Lee, Choong-Yeol Lee, Jin-Seok Lee, Chang-Eop Kim

**Affiliations:** ^1^ Department of Physiology, College of Korean Medicine, Gachon University, Seongnam, South Korea; ^2^ Department of Herbal Formula, College of Korean Medicine, Dongguk University, Goyang-si, South Korea; ^3^ Institute of Bioscience and Integrative Medicine, Daejeon Oriental Hospital of Daejeon University, Daejeon, South Korea

**Keywords:** flavonoids, non-alcoholic fatty liver disease, network pharmacology, network medicine, machine learning

## Abstract

Nonalcoholic fatty liver disease (NAFLD) is the most common type of chronic liver disease and lacks guaranteed pharmacological therapeutic options. In this study, we applied a network-based framework for comprehensively identifying candidate flavonoids for the prevention and/or treatment of NAFLD. Flavonoid-target interaction information was obtained from combining experimentally validated data and results obtained using a recently developed machine-learning model, AI-DTI. Flavonoids were then prioritized by calculating the network proximity between flavonoid targets and NAFLD-associated proteins. The preventive effects of the candidate flavonoids were evaluated using FFA-induced hepatic steatosis in HepG2 and AML12 cells. We reconstructed the flavonoid-target network and found that the number of re-covered compound-target interactions was significantly higher than the chance level. Proximity scores have successfully rediscovered flavonoids and their potential mechanisms that are reported to have therapeutic effects on NAFLD. Finally, we revealed that discovered candidates, particularly glycitin, significantly attenuated lipid accumulation and moderately inhibited intracellular reactive oxygen species production. We further confirmed the affinity of glycitin with the predicted target using molecular docking and found that glycitin targets are closely related to several proteins involved in lipid metabolism, inflammatory responses, and oxidative stress. The predicted network-level effects were validated at the levels of mRNA. In summary, our study offers and validates network-based methods for the identification of candidate flavonoids for NAFLD.

## 1 Introduction

Non-alcoholic fatty liver disease (NAFLD) is known to accumulate fat (particularly over 5%) in the liver tissue of patients who do not ingest excessive quantities of alcohol. It is a representative metabolic disorder in the liver, which presents a variety of conditions, including simple steatosis, steatohepatitis, liver fibrosis, and hepatocellular carcinoma (Anstee et al., 2019). Although the exact pathophysiological mechanism of NAFLD is not fully understood, the most accepted concept is considered as the “multiple-hits model,” which involves prevalent multiple parallel factors, including fat accumulation, inflammatory reactions, and oxidative stress (Buzzetti et al., 2016). Recent data show that 25% of general population has NAFLD, and the rate is much higher in high-income countries. (Mitra et al., 2020). Recently, NAFLD has become one of the most important medical issues worldwide; however, there has been no effective therapeutic approach. Thus, the development of anti-NAFLD effect-guaranteed therapeutics is urgently required.

Natural products, such as flavonoids, have frequently been investigated in NAFLD models and have shown beneficial effects in clinical and preclinical studies. A multiethnic clinical study found that the intake of flavonoids reduces the risk of NAFLD and assists in normalizing NAFLD status by attenuating the fatty liver index, serum aspartate aminotransferase, and alanine aminotransferase (Mazidi et al., 2019). In addition, narrative reviews suggested that the pharmacological properties of flavonoids against NAFLD are primarily exerted by acting on multiple targets involved in oxidative stress, inflammation, and lipid metabolism (Jadeja and Devkar, 2013; Qiu et al., 2018). Despite their therapeutic potential, the majority of flavonoids have not been identified for their NAFLD treatment potential due to the excessive cost and labor required to perform biochemical analysis. Therefore, there is still a pressing need for an alternative strategy to predict potential flavonoids with in-depth mechanisms for prevention and treatment of NAFLD.

A network medicine framework, based on the molecular interactions of comprehensive subcellular networks, has emerged as a promising platform for identifying rational drug target and novel indication (Guney et al., 2016; Gysi et al., 2021). The key finding of the framework is that the closer the targets of a compound are to disease proteins on a human protein-protein interaction (PPI) network, the more likely that the compound will affect the disease phenotype. A recent study also revealed that the framework can discover the therapeutic effects of polyphenols, suggesting the possibility of discovering potential natural products for NAFLD treatment (do Valle et al., 2021). However, a sufficient amount of compound-target interaction (CTI) information is an essential prerequisite for exploring the therapeutic potential of natural products using the developed framework. Unfortunately, the CTIs of natural products are currently largely unknown, making it difficult to fully apply network medicine frameworks to natural products.

One way to complement the limited coverage of the framework is to leverage machine-learning prediction methods. Using abundant chemical and biological data, machine-learning techniques have been successfully applied in various applications and archives, including CTI prediction (Luo et al., 2017; Wen et al., 2017; You et al., 2019; Chen et al., 2020; Huang et al., 2020). In particular, Lee et al. recently developed a state-of-the-art algorithm, AI-DTI, that predicts activatory and inhibitory targets based on mol2vec and genetically perturbed transcriptome (Lee et al., 2021). The model outperformed a previous model that predicted activatory and inhibitory targets, supporting the accuracy and reliability of the model. One of the key features of this model is that only the 2D structure of a small molecule is required for CTI prediction, highlighting the practical applicability of CTI prediction to natural products.

**FIGURE 1:**
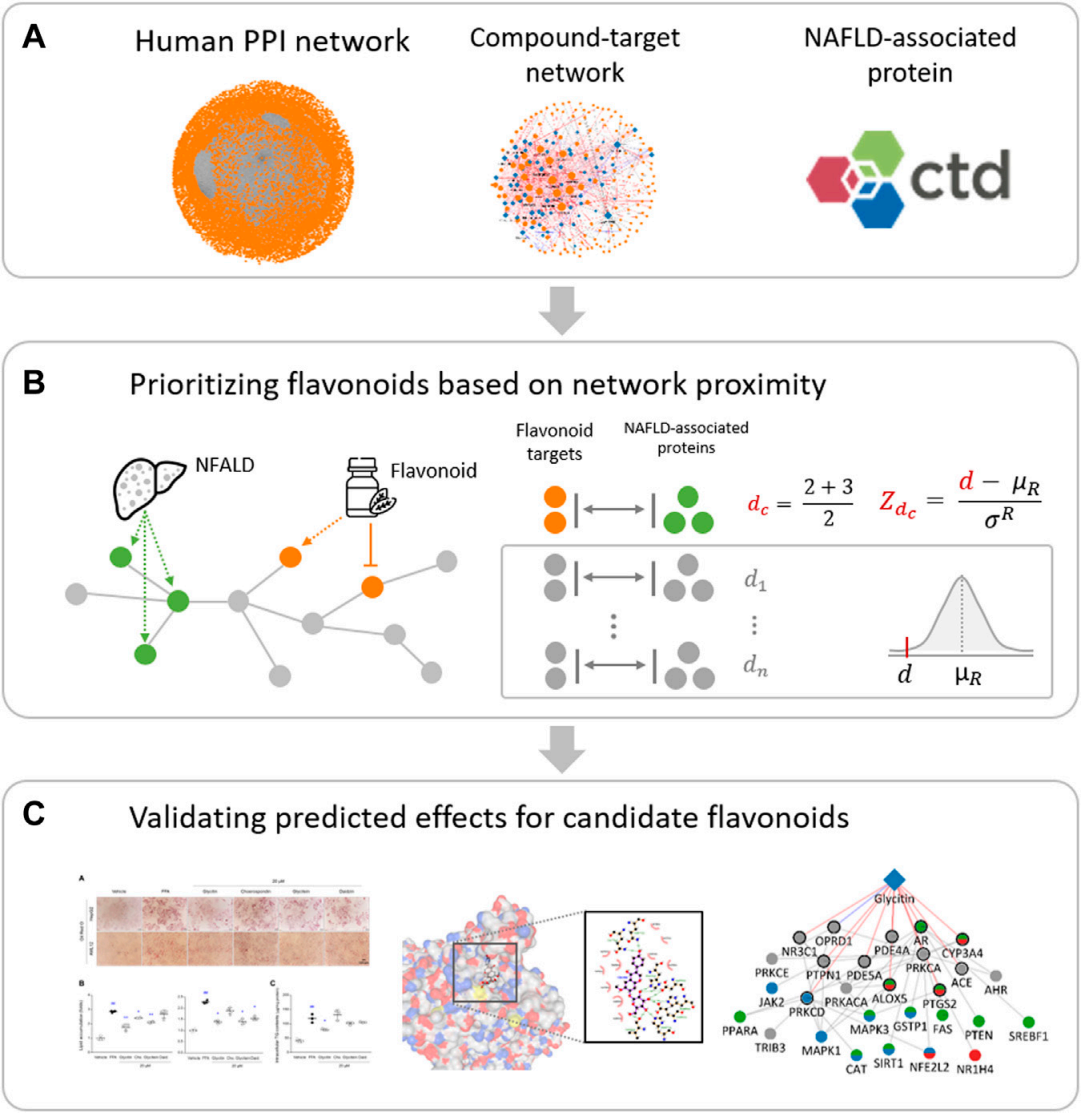
Integrated workflow for investigating candidate flavonoids and their potential mechanisms for NAFLD. **(A)** Data collection. Human PPI network, compound-target network of flavonoids, NAFLD-associated proteins were collected from various datasets and databases. **(B)** Prioritizing flavonoids based on network proximity. Average closest distance ( $d_{c}$ ) and its relative distance ( $Z_{d_{c}}$ ) were calculated to screen potential flavonoids for NAFLD treatment under the human PPI network. **(C)** Experimental validation. The preventive effects of candidate flavonoids against NAFLD were evaluated using FFA-induced *in vitro* model, molecular docking, and network analysis.

Here, we aimed to investigate potential flavonoids that exert beneficial effects on NAFLD by combining an AI-DTI model and the network medicine framework (Figure 1). Focusing on flavonoids, which have been widely studied for NAFLD, allowed us to comprehensively evaluate the reliability of the prediction results and lead to identifying more promising candidates. We assembled experimentally validated and predicted CTIs from public databases and AI-DTI, respectively, and then prioritized the potential flavonoids for prevention and/or treatment of NAFLD by calculating network proximity between flavonoid targets and NAFLD-associated proteins. We evaluated whether proximity measurements could rediscover known beneficial effects and their potential mechanisms in order to test the validity of the predicted results. Finally, the potential of the candidate flavonoids was evaluated using a free fatty acid (FFA)-induced HepG2 and AML12 cell models. Altogether, we believe that this study systematically revealed flavonoids that can be used against NAFLD, along with testable molecular mechanistic hypotheses.

## 2 Materials and Methods

### 2.1 Flavonoids Selection

Flavonoids were retrieved from the Phenol-Explorer database (version 3.6) (Rothwell et al., 2013). For the analysis, we only considered flavonoids that 1) could be mapped to PubChem IDs, and 2) where 2D structures in SMILES format were available. A quantitative estimate of drug-likeness (QED) (Bickerton et al., 2012) was calculated to select candidate flavonoids for bioactivity. Following this, 59 flavonoids were considered candidates with favorable pharmacokinetic properties that could be used in subsequent analyses. QED was calculated using the RDkit module in Python 3.7.

### 2.2 Predicting Flavonoid Targets

The potential target profiles of flavonoids were predicted by AI-DTI, a practically useful algorithm developed for predicting activatory and inhibitory targets of compounds (Lee et al., 2021). AI-DTI consists of two models that predict activatory or inhibitory CTIs. When an input query (drug-target pair) is received, AI-DTI transforms it into activatory and inhibitory DTI feature vectors, and then infers activatory and inhibitory interaction using each prediction model. The input features for the model are constructed by concatenating the vectors of compounds and targets derived from the mol2vec method (Jaeger et al., 2018) and a genetically perturbed transcriptome obtained from CMap (Subramanian et al., 2017). For activatory CTIs, the feature vector was represented as a concatenated form of the compound vector calculated by mol2vec and the representative vectors of activatory targets. For inhibitory CTIs, the feature vector was constructed as a concatenated form of the compound vector calculated by mol2vec and representative vectors of inhibitory targets. Each model is trained to discriminate between known and unknown CTIs based on a dataset consisting of the constructed input features and their labels. The trained model predicts the likelihood score that the compound would activate or inhibit the protein using the input vector of the compound and the target of interest. In this study, we used an AI-DTI model trained on the extended dataset with an optimized cascaded deep forest model trained on the extended dataset that can predict a wider target with best performance.

### 2.3 Network Pharmacological Analysis for Flavonoids

Network pharmacological analysis was conducted by constructing a compound-target network for flavonoids and analyzing the constructed network. A compound–target network is a bipartite network in which nodes are defined as compounds and targets, and the edges between compounds and targets are defined as CTIs. The compound-target network was constructed and visualized using Cytoscape (version 3.8.2) based on information regarding the compounds, targets, and their various interactions (Shannon et al., 2003). Functional annotation and Gene Ontology (GO) overrepresentation analyses were performed using the online analytical tool PANTHER (Protein ANalysis THrough Evolutionary Relationships; http://www.pantherdb.org, v.14.0) (Mi et al., 2019). PANTHER is widely used as a comprehensive resource for gene function classification and genome-wide data analysis. Fisher’s exact tests with the Benjamini-Hochberg false discovery rate correction were employed to determine the significance of GO terms in the biological process category of the *Homo sapiens* genome.

### 2.4 Non-Alcoholic Fatty Liver Disease-Associated Proteins

NAFLD-associated proteins were obtained from the Comparative Toxicogenomics Database (CTD) and the literature. CTD is a publicly available database that aims to advance the understanding of the effects of environmental exposure on human health, providing manually curated information, such as disease-gene associations (Davis et al., 2019). Among the disease-gene associations, we selected associations labeled as “Marker/Mechanism” and “Therapeutic”. We manually added 14 additional NAFLD-associated proteins from the literatures (Millar et al., 2006; Tariq et al., 2014; Wu W. et al., 2018; Yu et al., 2018). To identify biological functions at the process level, proteins were grouped as follows: lipid metabolism (lipid metabolic process, GO: 0006629), inflammation (regulation of inflammatory response, GO: 0050727), oxidative stress (response to oxidative stress, GO: 0006979), and others. In this study, 85 proteins related to various biological processes were identified as NAFLD-related proteins (Supplementary Table S1).

### 2.5 Human Protein-Protein Interaction Network

The human PPI network is a set of PPIs that occur in human cells. The PPI network used in this study was obtained from the data built by do Valle et al. (2021). Briefly, they assembled the human interactome from 16 databases containing six different types of PPIs: 1) binary PPIs tested by high-throughput yeast two-hybrid experiments (Rolland et al., 2014); 2) kinase–substrate interactions from literature-derived low-throughput and high-throughput experiments from Kinome NetworkX (Cheng et al., 2014), Human Protein Resource Database (HPRD) (Keshava Prasad et al., 2009) and PhosphositePlus (Hornbeck et al., 2012); 3) carefully literature-curated PPIs identified by affinity purification followed by mass spectrometry (AP-MS) and from literature-derived low-throughput experiments from InWeb (Li et al., 2016), BioGRID (Chatr-Aryamontri et al., 2017), PINA (Cowley et al., 2012), MINT (Peri et al., 2004), IntAct (Orchard et al., 2014) and InnateDB (Breuer et al., 2013); 4) high-quality PPIs from three-dimensional protein structures reported in Instruct (Meyer et al., 2013), Interactome3D (Mosca et al., 2013), and INSIDER (Meyer et al., 2018); 5) signaling networks from literature-derived low-throughput experiments as annotated in SignaLink2.0 (Fazekas et al., 2013); and 6) protein complexes from BioPlex2.0 (Huttlin et al., 2017). The genes were mapped to their Entrez IDs based on the National Center for Biotechnology Information (NCBI) database and their official gene symbols. The constructed network included 351,444 PPIs, connecting 17,706 unique proteins.

### 2.6 Network Proximity Calculation Between Flavonoid Targets and Non-Alcoholic Fatty Liver Disease Proteins

The proximity of NAFLD-associated proteins and flavonoids was assessed using a distance metric proposed by Guney et al. (2016), considering the shortest path length between the targets of the compound and the disease protein.

First, the average closest distance $d_{c}\left(S,T\right)$ between NAFLD-associated proteins and flavonoid targets is defined as follows:

$$d_{c}\left(S,T\right)=\;\frac{1}{\left|\left|T\right|\right|}\sum_{t\in T}\min_{s\in S}d\left(s,t\right)\;$$ (1)

S denotes a set of NAFLD-associated proteins, T denotes the set of flavonoid targets, and d(s,t) denotes the shortest path length between nodes s and t in the network. A relative distance metric ( $Z_{d_{c}}$ ) was then calculated by comparing the $d_{c}\left(S,T\right)$ to a reference distribution describing random expectations. The reference distribution is constructed by iteratively calculating the expected distances between two randomly selected groups of proteins matching the size and degrees of NAFLD–associated proteins and flavonoid targets in the network. The relative distance $Z_{d_{c}}$ is defined as follows:

$$Z_{d_{c}}=\frac{d-\;\mu_{d_{c}\left(S,T\right)}}{\sigma_{d_{c}\left(S,\;T\right)}}$$ (2)

$\mu_{c\left(S,T\right)}$ denotes the mean and $\sigma_{c\left(S,T\right)}$ denotes standard deviation of the reference distribution, respectively. The closest and relative distances were calculated in python 3.7 using a module shared by Guney et al.

### 2.7 Molecular Docking

The molecular docking method was used to study the binding affinities and conformations of glycitin and its predicted targets. The web server CB-Dock was used to perform molecular docking simulations (Liu et al., 2019). The PDB (Protein Data Bank) formats of proteins and ligand files in SDF formats were derived from the PDB (http://www.rcsb.org) (Burley et al., 2017) and PubChem (http://www.ncbi.nlm.gov/pccompond) (Kim et al., 2021), respectively. These files were uploaded and submitted to the CB-Dock server. The result table lists the vina scores, cavity sizes, docking centers, and sizes of the predicted cavities. Once a ligand in the table is selected, the structure in the interactive 3D graphics is visualized. For cross-validation, the COACH-D server (https://yanglab.nankai.edu.cn/COACH-D/) (Wu Q. et al., 2018) was also applied to predict putative ligand binding sites and binding energies. Ligplot software was used for 2D visualization of the interactions between proteins and a ligand.

### 2.8 Experimental Methods

#### 2.8.1 Cell Culture

HepG2 cell line (Korean Cell Line Bank, Seoul, Republic of Korea) and RAW264.7 cell line were cultured in Dulbecco’s Modified Eagle’s Medium (WelGENE Inc., Seoul, Republic of Korea) with 10% fetal bovine serum (WelGENE Inc.) and 1% antibiotic-antimycotic solution (WelGENE Inc.). Cells were maintained in a humidified incubator at 37°C in 5% CO_2_. AML12 cell line (ATCC, VA. United States) was maintained in DMEM/F12 (GIBCO/Thermo Fisher, NY, United States) supplemented with 10% FBS, 10 μg/ml insulin, 5 μg/ml transferrin, 7 ng/ml selenium, 40 ng/ml dexamethasone and 1% of antibiotic-antimycotic solution (GIBCO or WelGENE). Cells were maintained in a humidified incubator at 37°C in 5% CO_2_.

#### 2.8.2 Cell Viability Assay

HepG2 cells (7 × 10^3^ cells/well) were seeded in 96-well microplates and incubated for 12 h. Cells were treated with 2, 20, or 200 μM of glycitin, choerospondin, glycitein, and daidzin (MedChemExpress, NJ, United States) for 24 h. Cell viability was measured using the commercially available reagent Cell Counting Kit-8 (EZ-Cytox, DoGen, Republic of Korea) according to the manufacturer’s instructions. Absorbance was measured using a UV spectrophotometer at 450 nm (Molecular Devices, CA, United States).

#### 2.8.3 Oil Red O Staining Assay

Based on the cell viability results, HepG2 cells and AML12 cells (2 × 10^5^ cells/well) seeded in 6-well plates were treated with 200 μM of FFA mixture (ratio of 2: 1, oleic acid: palmitic acid, dissolved in 1% bovine serum albumin) for 24 h after pretreatment with 20 μM of four single compounds (glycitin, choerospondin, glycitein, and daidzin). The cells were then fixed with 10% formaldehyde for 30 min at room temperature. After washing with phosphate-buffered saline, cells were stained with Oil Red O working solution (3 mg/ml in 60% isopropanol) for 10 min. Lipid accumulation was detected using optical microscopy. Moreover, intracellular lipid content was quantified by disrupting the cells with 100% isopropanol. Absorbance was measured using a UV spectrophotometer at 492 nm (Molecular Devices).

#### 2.8.4 Triglyceride Detection Assay

Under the same cell culture conditions as the Oil Red O staining assay, intracellular TG levels were measured using an enzymatic detection kit (Asan Pharmaceuticals, Seoul, Republic of Korea). Total protein concentration was measured using a bicinchoninic acid protein assay kit (BCA1 and B9643, Sigma-Aldrich, MO, United States). The absorbance was measured using a UV spectrophotometer at 562 nm (Molecular Devices).

#### 2.8.5 Reactive Oxygen Species Detection Assay

Under the same cell culture conditions as the Oil Red O staining assay, intracellular ROS levels were measured using a 2′, 7′-dichlorofluorescein diacetate (H_2_DCFDA) fluorescent probe. Briefly, cells were incubated with 10 μM DCFH-DA for 30 min at 37°C in the dark. Intracellular ROS production was measured using an Axiophot microscope (Carl Zeiss, Jena, Germany).

#### 2.8.6 Nitric Oxide Detection Assay

RAW264.7 cells (2 × 10^4^ cells/well) seeded in 96-well plates were treated with 100 ng/ml of lipopolysaccharide (LPS) for 24 h after pretreatment with 20 μM of four single compounds (glycitin, choerospondin, glycitein, and daidzin). The supernatants were incubated with equal amount of Griess reagent (1% sulfanilamide/0.1% N-(1-naphthyl)-ethylenediamine dihydrochloride/2.5% H_3_PO_4_). After incubation for 15 min at 37°C, absorbance was measured using a UV spectrophotometer at 405 nm (Molecular Devices).

#### 2.8.7 Tumor Necrosis Factor-α Detection Assay

Under the same cell culture conditions as the NO detection assay, the proinflammatory cytokine TNF-α level of supernatants was measured using a commercially available enzyme immunoassay (EIA) kit for TNF-α (BD Biosciences, San jose, CA, United States). The absorbance was measured using a UV spectrophotometer at 450 nm (Molecular Devices).

#### 2.8.8 Quantitative Real-Time PCR

Under the same cell culture conditions as the Oil Red O staining assay, total mRNA was extracted using QIAzol reagent (Qiagen, CA, United States). After synthesis of cDNA using a High-Capacity cDNA Reverse Transcription Kit (Ambion, Austin, TX, United States), real-time PCR was performed using SYBR Green PCR Master Mix (Applied Biosystems; Foster City, CA, United States). PCR amplification was performed using a Rotor-Gene Q (Qiagen, Hilden, Germany) with standard protocol. The quantitative cycle threshold value of each gene was normalized with that of GAPDH. Information of the primer sequences is summarized in Supplementary Table S2.

### 2.9 Statistical Analysis

Statistical analyses were performed using Python (version 3.7) with SciPy module (version 1.7.1). For the two-sample test, Shapiro–Wilk test was used to assess whether the data were normally distributed. A two-tailed Student’s t test was then applied to compare unpaired two groups with normality. When the normality was rejected, the Mann-Whitney U test was applied. For the multiple comparison test, Shapiro–Wilk test was used to assess whether the data were normally distributed. To compare unpaired multiple sample groups, one-way analysis of variance (ANOVA) followed by Dunnett’s test was used for the dataset with normality. When the normality was rejected, Kruskal-Wallis ANOVA followed by Dunn’s post hoc test was applied. All statistical significance was set at *p* < 0.05.

## 3 Results

### 3.1 Flavonoid Selection and Subclass Analysis

**FIGURE 2:**
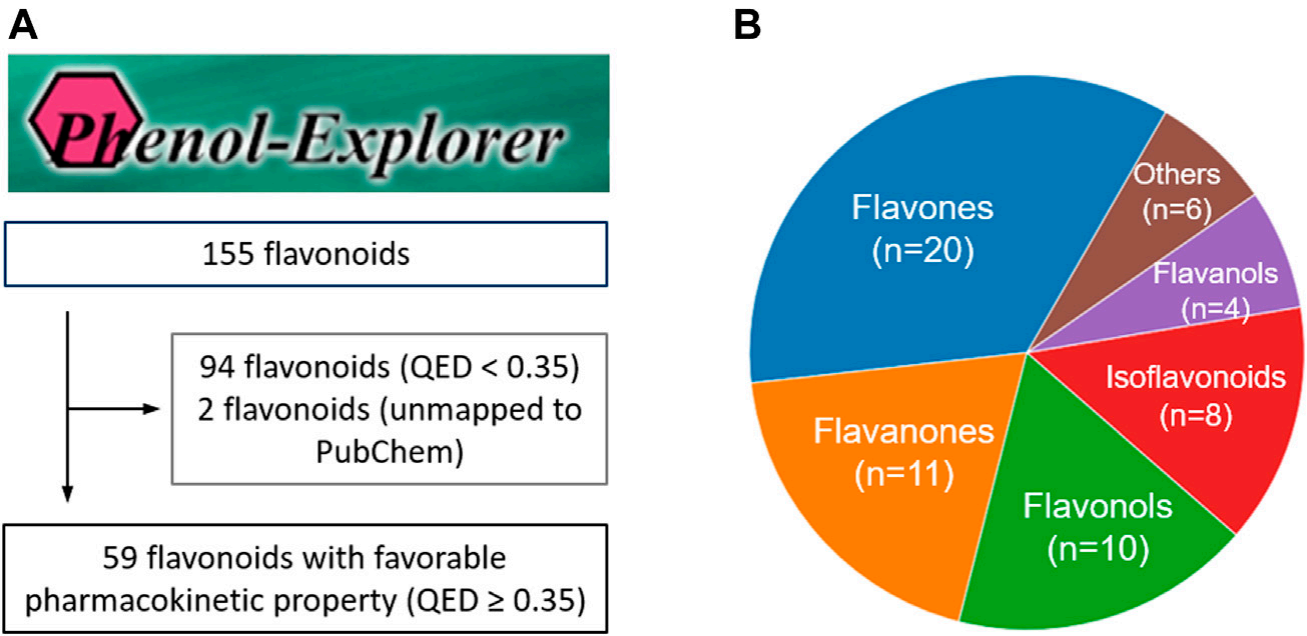
Selection process for flavonoids evaluated in this study and their chemical distribution. **(A)** The flowchart of selecting the flavonoids **(B)** Distribution of flavonoids across its subclasses.

We initially identified 155 flavonoids from Phenol-Explorer. We selected flavonoids that could be mapped to a valid PubChem ID and had a QED score ≥0.35 in order to consider validity and bioavailability, respectively. This threshold represents the average QED value of FDA-approved drugs, and compounds with QED values above the threshold are considered to have favorable pharmacokinetic properties. As a result, 59 flavonoids were selected and included in our study (Figure 2A and Supplementary Table S3). To describe their chemical diversity, we visualized the subclass distribution of flavonoids. The results showed that the selected flavonoids were distributed across nine subclasses. Among the subclasses, flavones, flavanones, and flavonols were the top three subclasses with 20, 11, and 10 compounds, respectively (Figure 2B).

### 3.2 Construction of Drug-Target Network for Flavonoids

**FIGURE 3:**
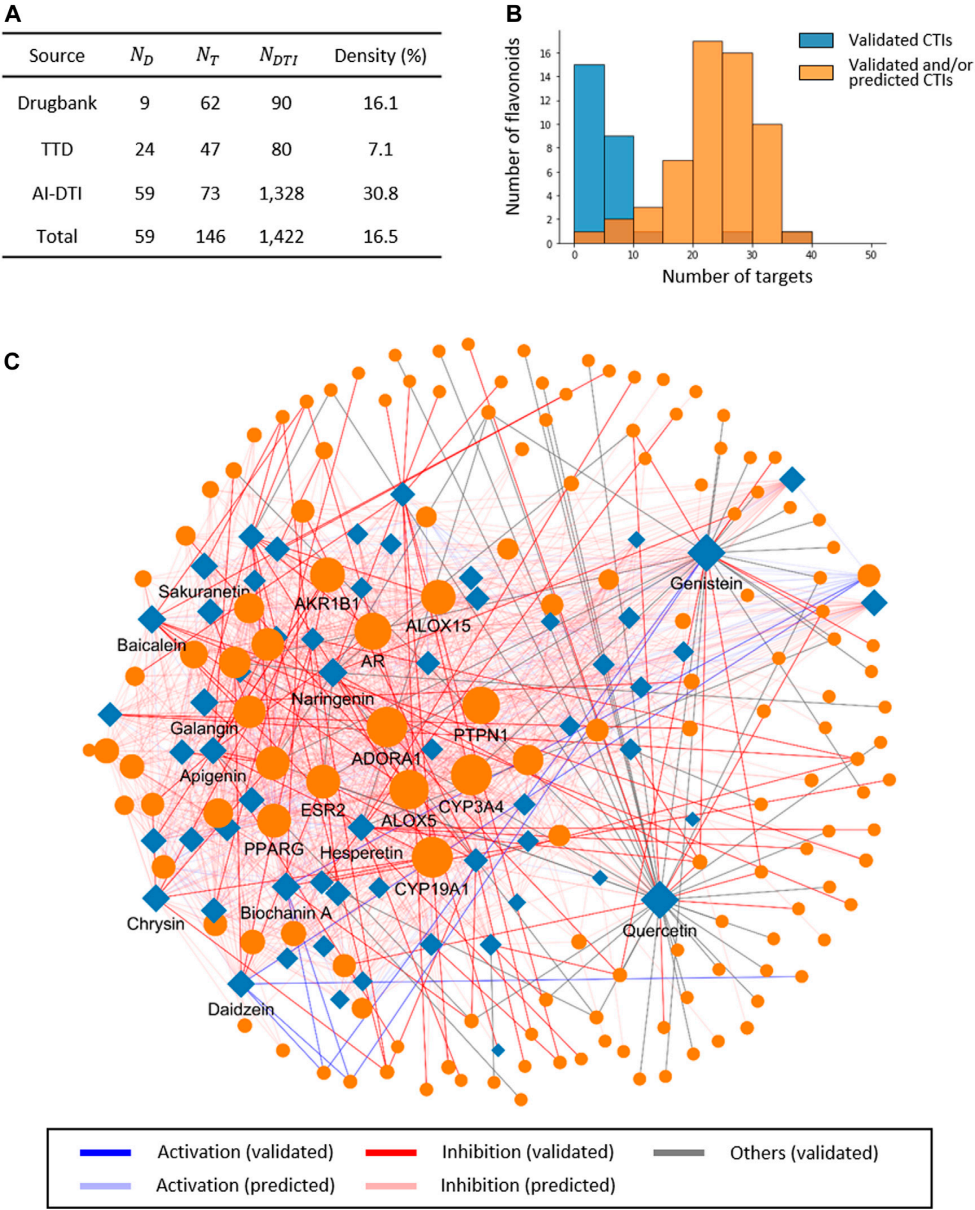
A compound-target network for selected flavonoids and its property. **(A)** Statistics of experimentally validated and/or predicted compound-target interactions (CTIs) for flavonoids. DrugBank and TTD contain experimentally validated DTIs, and AI-DTI is employed to predict the activity-inhibitory target of flavonoids. **(B)** Distribution of the number of flavonoid targets. **(C)** Compound-target network for flavonoids. Circles and diamonds denote protein targets and compounds, respectively.

We first retrieved 168 validated CTIs between 27 flavonoids and 100 protein targets from the DrugBank (version 5.1.8) database and Therapeutic Target Database (TTD, version 2.0). We discovered that the percentage of flavonoids with more than five known targets was only 20% (12/59), requiring additional CTI information for subsequent analysis (Figure 3B). Therefore, we utilized our recently developed algorithm, AI-DTI, to predict activatory and inhibitory targets of selected flavonoids. We constructed an input vector for all predictable flavonoid and protein target pairs, and then predicted the likelihood score of the CTI using a pretrained model. Compound-target pairs were considered to activate or inhibit if their prediction score was >0.82, which exhibited a false discovery rate of 1% in the default dataset. As a result, we additionally secured 124 activatory CTIs and 1,130 inhibitory CTIs between 59 selected flavonoids and 73 protein targets (Figure 3A and Supplementary Table S4).

We tested the reliability of the predicted results by comparing whether the overlapped number of CTIs between the experimentally validated results and predicted results was higher than the values in the null distribution. The values of the null distribution were obtained by randomly selecting the potential combinations of flavonoids and predictable targets in AI-DTI and then repeatedly calculating the number of overlapped CTIs between validated results and selected results. We found that AI-DTI successfully recovered 11 validated CTIs that did not appear in the training dataset. The number of recovered CTIs between the validated results and predicted results was significantly higher than the chance level (*p* < 0.001, hypergeometric test), which supports the reliability of the predicted results of our model for selected flavonoids.

We constructed and visualized a compound-target network between the selected flavonoids and the target protein using assembled experimentally validated and predicted CTIs (Figure 3C). The network consisted of 205 nodes and 1,422 edges, in which nodes denote the selected compounds or protein targets (59 and 146, respectively), and edges denote activatory, inhibitory, or other/unknown CTIs (133, 1228, and 61, respectively). We revealed that the average number of targets for flavonoids was 23.7, and the majority of the flavonoids had more than 10 targets, except for three flavonoids (Figure 3B). These results show that AI-DTI can provide accurate and sufficient CTI information for subsequent analyses.

### 3.3 Predicting Candidate Flavonoids for Non-Alcoholic Fatty Liver Disease Based on Network Proximity

Next, we attempted to identify candidate flavonoids that exert beneficial effects on NAFLD by employing a network medicine framework. We calculated the network proximity between flavonoid targets and NAFLD-associated 85 proteins using the closest measure, $d_{c}$ and $Z_{d_{c}}$ , representing the average shortest path length and its relative distance between each flavonoid target and the nearest disease protein, respectively (Figure 1B, see Materials and Methods for details). The measured proximity and direct interactions between the flavonoids and NAFLD-associated proteins are summarized in Supplementary Table S5. For example, daidzein and daidzin, an isoflavone phytoestrogen found in soy, and its metabolites are produced by human intestinal microflora. An *in vivo* study found anti-steatotic effects of daidzein through direct regulation of hepatic *de novo* lipogenesis and insulin signaling, and the indirect control of adiposity and adipocytokines by altering adipocyte metabolism. We found that daidzin was more proximal to NAFLD-associated proteins than any other flavonoid ( $d_{c}$ = 1.1, $Z_{d_{c}}$ = −4.86). We extended our search range to assess whether the top-predicted flavonoids and their metabolic byproducts have reported beneficial effects on NAFLD. Our results also revealed that the majority of flavonoids proximal to NAFLD-associated proteins have therapeutic effects on NAFLD, indicating that proximity score successfully rediscovered the known therapeutic effect of flavonoids on NAFLD (Table 1).

**TABLE 1:** Top network-predicted candidate flavonoids for NAFLD with available literature-derived evidence.

$d_{c}$	$Z_{d_{c}}$	PubChem ID	Name	Sub-class	References (PMID)
1.10	−4.86	107971	Daidzin	Isoflavonoids	21157426^#^
1.12	−4.32	187808	Glycitin	Isoflavonoids	NA
1.17	−3.92	156155	6″-O-Acetyldaidzin	Isoflavonoids	NA
1.21	−3.73	513197	Isoxanthohumol	Flavanones	26976708^#^
1.21	−3.14	10185	Dihydroquercetin	Dihydroflavonols	33325949
1.21	−3.71	92794	Naringenin 7-O-glucoside	Flavanones	33234364^#^
1.21	−3.71	470791	Choerospondin	Flavanones	NA
1.25	−3.37	72344	Nobiletin	Flavones	33096235
1.25	−3.28	155094	6-Prenylnaringenin	Flavanones	26976708^#^
1.25	−3.28	509245	8-Prenylnaringenin	Flavanones	26976708^#^
1.25	−3.23	5281222	Butein	Chalcones	22722906

^#^Previous evidence of its metabolites.

**FIGURE 4:**
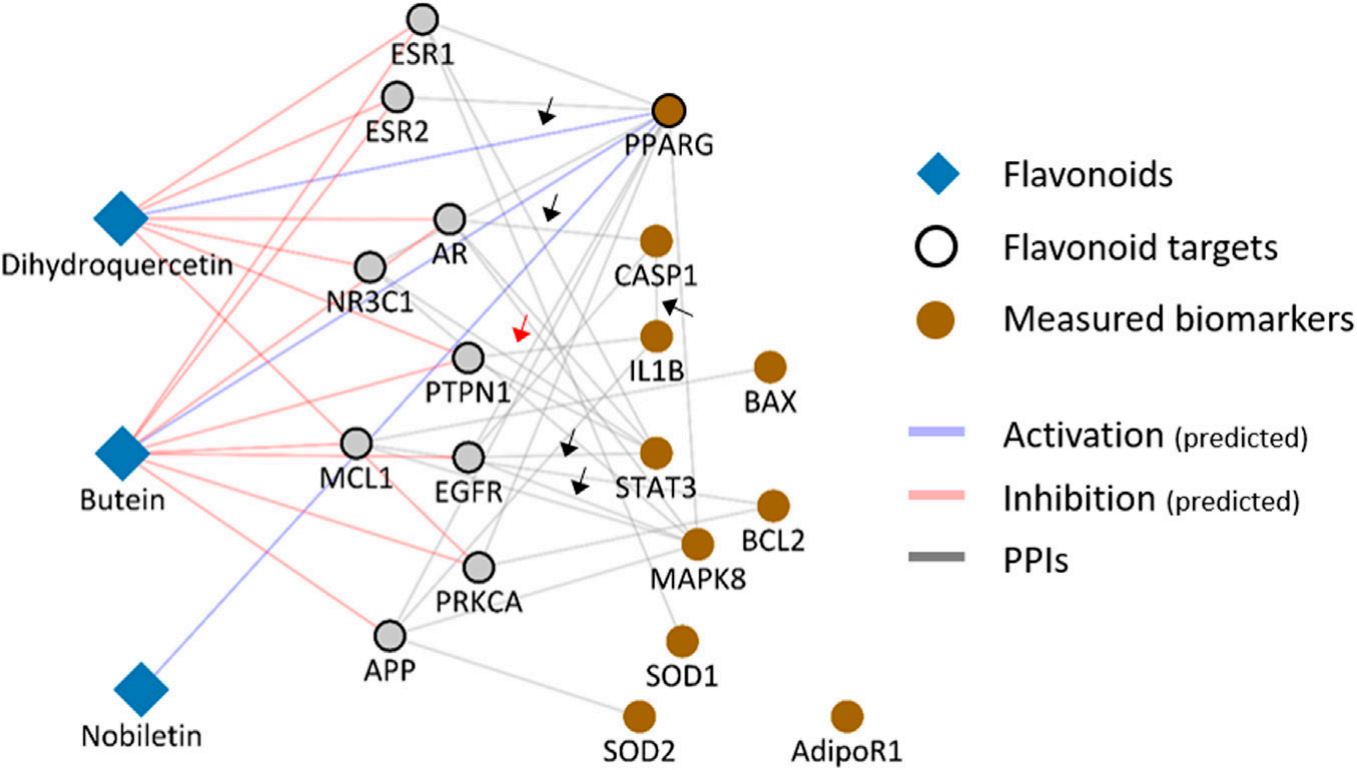
A compound-protein network between selected flavonoids and NAFLD-associated proteins. A network including the interaction between flavonoid targets and NAFLD-associated proteins was constructed to elucidate the mechanisms of dihydroquercetin, butein, and nobiletin. Black and red arrows indicate interactions consistent or inconsistent with the inferred mechanism of flavonoids against NAFLD, respectively.

To test the mechanistic interpretability of the framework, we evaluated whether the mechanisms of flavonoids could be explained at the network level. We considered three flavonoids, dihydroquercetin, nobliletin, and butein, which are highly ranked in proximity measure, and have evidence reported for its native molecule itself. We visualized networks focusing on selected flavonoids and their protein targets and biomarkers whose expression was measured in previous studies (Figure 4). We then explored whether the association between the target of flavonoids and the measured biomarker could explain the results of previous reports.

Dihydroquercetin, also called taxifolin, was reported to ameliorate high-fat diet feeding plus acute ethanol-binding induced steatohepatitis by upregulating PPARγ levels and suppressing the expression of interleukin (IL)-1β and caspase-1 (Zhan et al., 2021). Our results showed that dihydroquercetin activates PPARG, which supports the notion that upregulated PPARγ expression can be caused by the direct effect of dihydroquercetin. In addition, we infer that the inhibitory effects of dihydroquercetin on caspase-1 and IL-1β can be derived from the inhibitory effects of dihydroquercetin on the androgen acceptor. This hypothesis is supported by a previous study showing that the androgen receptor is a promising regulator of caspase-1 activity, which is responsible for the subsequent activation of pro-inflammatory cytokines, including IL-1β (Duez and Pourcet, 2021). Alternatively, butein exerts its antiproliferative and proapoptotic effects on NAFLD by suppressing STAT3 and JNK signaling (Moon et al., 2010; Rajendran et al., 2011). The constructed network showed that STAT3 and MAPK8 (JNK1) interacted closely with the butein target EGFR. Moreover, considering that EGFR is an upstream regulator of STAT3 and MPAK8 (JNK1), we can infer the potential mechanisms by which butane affects STAT3 and MAPK by regulating EGFR. Taken together, we found that molecular interactions between the flavonoid target and the measured biomarker provide potential network-level mechanisms for certain flavonoids.

In contrast, we found that associations between certain flavonoid targets and NAFLD-associated proteins does not aid in the interpretation of mechanisms. For example, previous studies have reported that dihydroquercetin inhibits the expression of IL-1B, which can lead to a hypothesis that its effect is exerted by the inhibitory effect of dihydroquercetin on interacting PTPN1, which interact with IL-1β. However, a previous report revealed that PTPN1 inhibition rather further increases the effectiveness of inflammatory cytokines, including IL-1β (Chen et al., 2015). Furthermore, we could not identify any direct neighbors between the target of noviletin and previously measured biomarkers. These results indicate that the molecular interactions between flavonoid targets and measured biomarkers should be meticulously interpreted, considering the disease and model-specific contexts.

### 3.4 Evaluating Preventive Effects of Flavonoids Using Free Fatty Acid-Induced Hepatic Steatosis Model

Based on the results from the proximity distances, we further evaluated whether unknown flavonoids, whose targets are proximal to NAFLD-associated proteins, could exhibit beneficial effects on NAFLD. We considered the following four flavonoids: glycitin and choerospondin, which are unreported and commercially available flavonoids that are proximal to NAFLD-associated proteins, and glycitein (metabolites of glycitin), and daidzin (the most proximal flavonoids with reported evidence). To ensure appropriate dose of four flavonoids (0–200 μM), the cell viability was evaluated using a cell counting kit-8 (CCK8) assays. Except highest treated dose at 200 μM, the flavonoid treatments didn’t show cytotoxicity in HepG2 cells (Supplementary Figure 1A). Therefore, further investigations for evaluating anti-NAFLD activity were performed using a single dose (20 μM) of four flavonoids.

**FIGURE 5:**
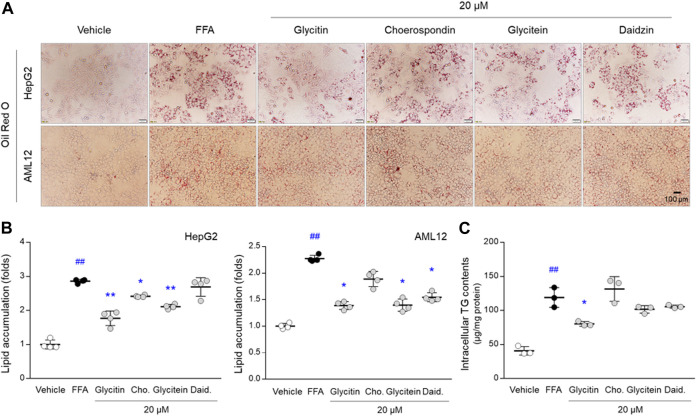
Effects of four isoflavones against NAFLD conditions in both HepG2 and AML12 cells. **(A)** Hepatic lipid accumulations and ROS productions were determined using Oil Red O staining and DCFH-DA assay, respectively. **(B)** Intracellular lipid accumulations in HepG2 and AML12 cells and **(C)** TG contents in HepG2 cells were quantified. Data are expressed as the mean ± SD (*n* = 3 or 4). ^##^ *p* < 0.01 compared to the vehicle-treated cells, and ^∗^ *p* < 0.05 and ^∗∗^ *p* < 0.01 compared to the cells exposed to FFAs.

To evaluate the beneficial effects of flavonoids against NAFLD, we adopted an FFA-induced hepatic steatosis cell model, which is commonly used to generate a cellular model of NAFLD (Müller and Sturla, 2019). For our experimental purpose, instead of palmitic acid that induces lipotoxicity, the FFA mixture (ratio of 2: 1, oleic acid: palmitic acid) was used. The FFAs predominantly induced NAFLD-like *in vitro* conditions in HepG2 cells, as evidenced by increases in Oil Red O histological observations (approximately 3-folds) and TG contents (approximately 3.1-folds). These NAFLD-like conditions were slightly or dramatically prevented by flavonoids, in particular, pretreatment with glycitin significantly inhibited the elevations of lipid accumulation and TG contents (a reduction of 39 and 33%, respectively, *p* < 0.05 or *p* < 0.01, Figures 5A–C). In addition, these anti-NAFLD properties were re-validated in a normal murine hepatocyte AML12 cells (Figures 5A,B). Taken together, these results indicate that the proximity scores are reliable traits for predicting novel candidates for preventing NAFLD progression.

**FIGURE 6:**
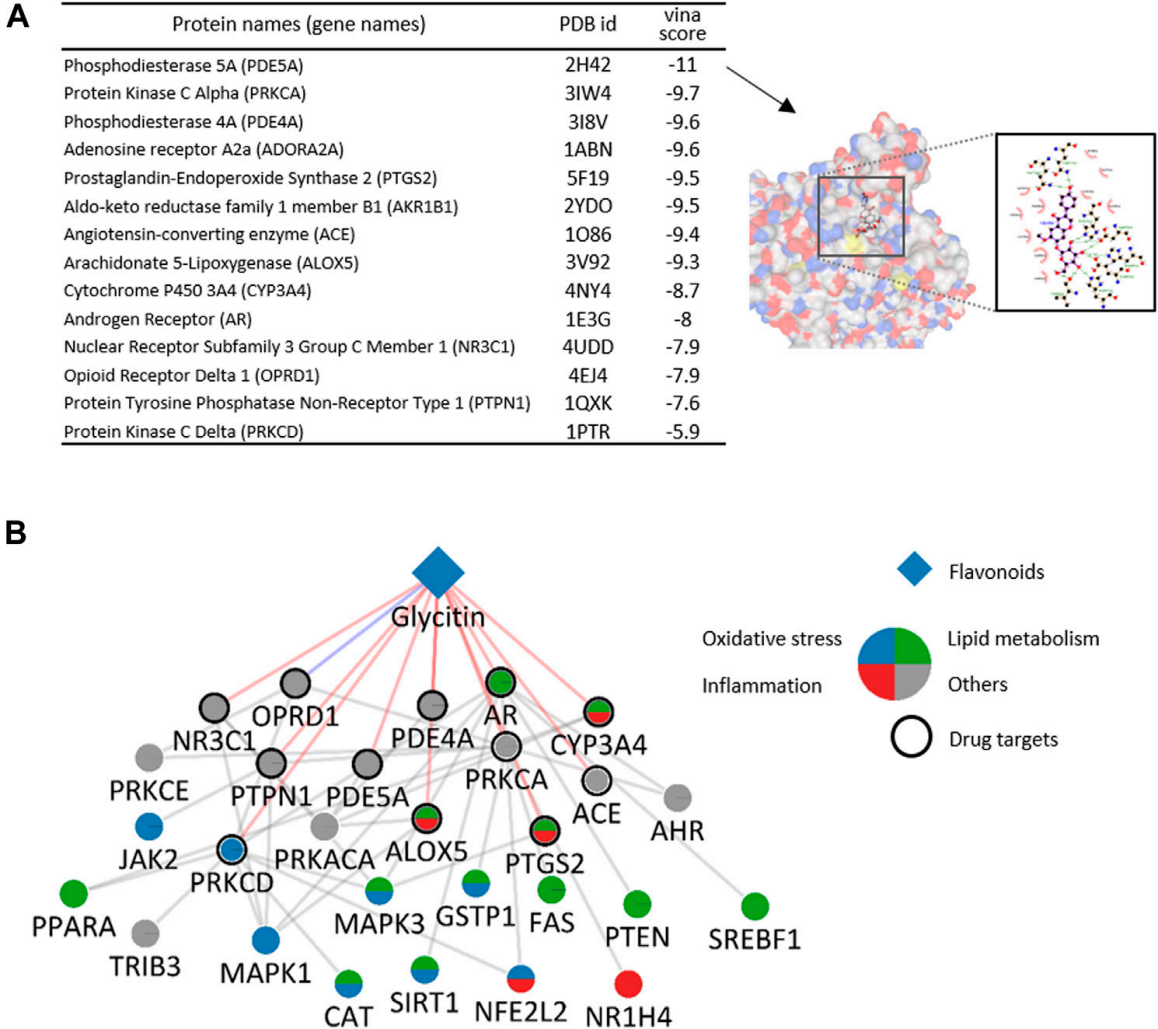
Molecular docking validation and potential mechanism of glycitin. **(A)** Molecular docking results between glycitin and its predicted target and its representative example. **(B)** Discovered target-NAFLD associated protein network for glycitin. A diamond denotes a flavonoid and circles denote protein targets. The border and color of the circle denote the predicted target or related process-level function, respectively.

We further investigated the potential mechanisms focusing on glycitin, and all the identified targets were predicted using AI-DTI. Reliability of predicted interactions was firstly evaluated by analyzing molecular docking potentials. Briefly, the structure of glycitin was uploaded to CB-dock (Liu et al., 2019) along with 14 predicted targets for which the structure was available in PDB. For each process, blind docking was carried out to detect suitable binding sites for glycitin and calculate the vina score, which is an estimate of the logarithm of the free binding energy. The results showed that the mean value of vina scores was −8.8 kcal/mol, and all vina scores between glycitin and the predicted protein target were less than −5 kcal/mol (Figure 6A). A cross-validation study was further performed using another docking web server, COACH-D (Wu Q. et al., 2018). The binding affinity between glycitin and the expected target ranged from −5.8 to −12.9 kcal/mol, which is consistent with the above results (Supplementary Table S6). We visualized the molecular interaction between glycitin and PDE5A, which had the lowest vina score. Our results show that glycitin exhibits a strong binding affinity to the predicted target, which supports the reliability of the predicted results.

We then conducted an overrepresentation test and network analysis to identify the potential mechanisms of glycitin at the level of biological processes and molecular interactions. The enrichment results based on GO showed that seven and six targets of glycitin were significantly involved in the regulation of lipid metabolic processes and inflammatory responses, respectively (approximately more than 5-folds to chance level, *p* < 0.001). This association indicates that the mechanisms of glycitin involve lipid metabolism and inflammation, which are key regulators of the pathogenesis and progression of NAFLD (Buzzetti et al., 2016). We also visualized the molecular interactions between the target of glycitin and NAFLD-associated protein. We found that glycitin targets interact with NAFLD-associated proteins associated with various functions, including oxidative stress (Figure 6B). The close interaction between them indicates that the antioxidant capacity of glycitin may be exerted by regulating the function of these proteins *via* the cellular network. These results indicate that the anti-NAFLD effect of glycitin may be exerted by regulating the functions of various proteins related to metabolism, oxidative stress, and inflammation at the biological process and network level.

**FIGURE 7:**
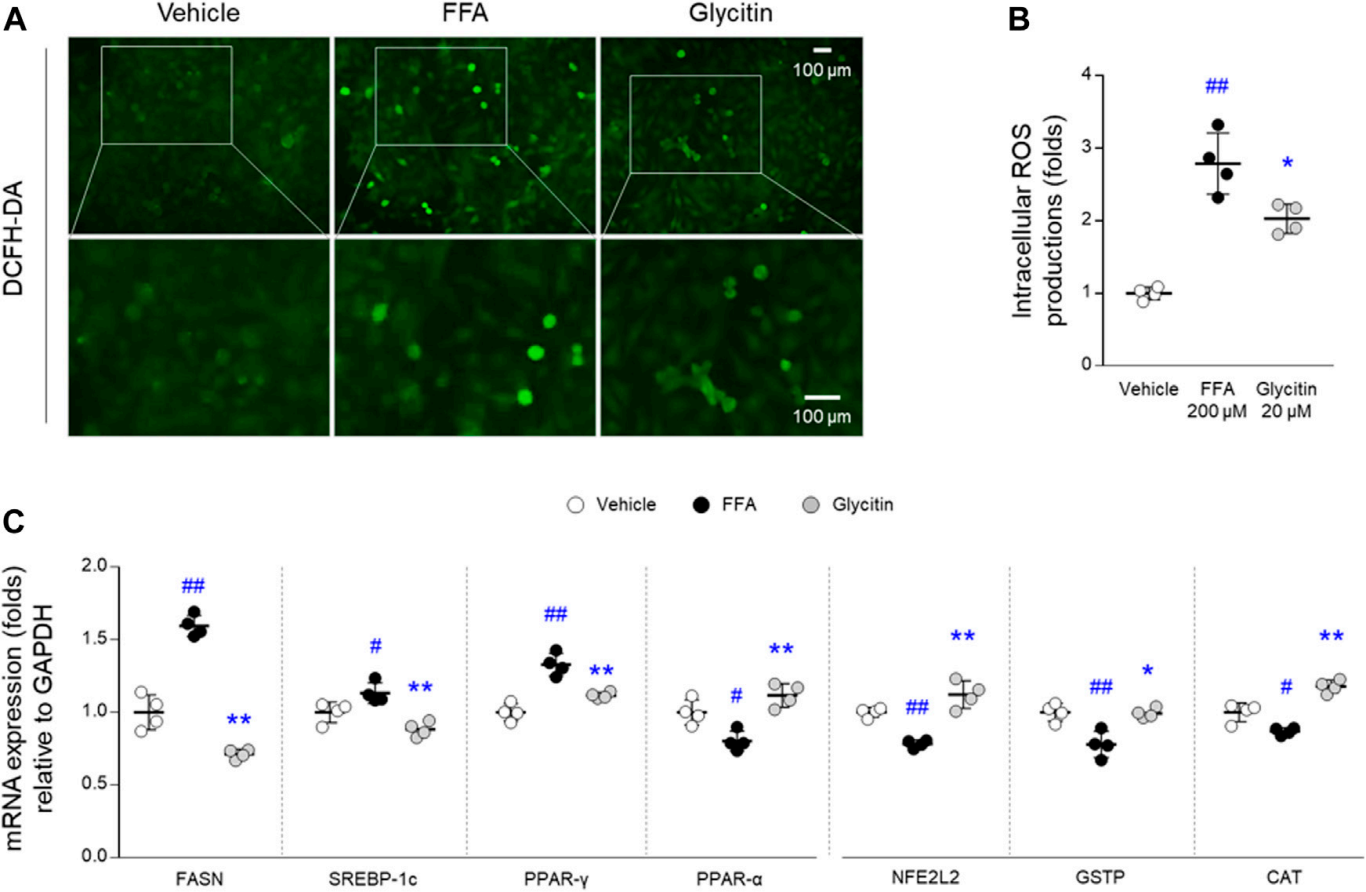
Experimental investigating potential mechanism of glycitin. **(A)** Intracellular ROS production was determined using DCFH-DA fluorescence assay, and **(B)** it was quantified in HepG2 cells. **(C)** The mRNA expressions of lipid metabolism- and antioxidant-related genes were measured using quantitative real-time PCR method in HepG2 cells. Data are expressed as the mean ± SD (*n* = 4). ^##^ *p* < 0.05 and ^##^ *p* < 0.01 compared to the vehicle-treated cells, and ^∗^ *p* < 0.05 and ^∗∗^ *p* < 0.01 compared to the cells exposed to FFA.

For the experimental verification of above predicted molecular interactions, we explored the mRNA gene expression in each of lipid metabolism, oxidative stress and inflammation using quantitative PCR method. Not surprisingly, the altered mRNA levels of FASN, SREBP-1c, PPAR-γ and α were prevented by glycitin pre-treatment (*p* < 0.01 for all lipid metabolism-related genes, Figure 7C). These results may be contributed by strong antioxidant capacity of glycitin, as shown in remarkable improvements against intracellular ROS overproduction arisen from FFA (*p* < 0.05, Figures 7A,B). Furthermore, glycitin exerted more up-regulations in mRNA levels of the NFE2L2, GSTP, and CAT (*p* < 0.05 or *p* < 0.01, respectively; Figure 7C), and their levels were tended to increase than vehicle-treated cells.

### 3.5 Evaluating Preventive Effects of Flavonoids Using Lipopolysaccharid-Induced Inflammation Model

According to AI-DTI predictions, we additionally investigated the anti-inflammatory properties of flavonoids using LPS-induced inflammatory macrophage model. In RAW264.7 macrophage cell, the LPS significantly increased the levels of NO, TNF-α and IL-10 (*p* < 0.01 for all parameters). The increases in both NO and TNF-α levels were considerably attenuated by flavonoids (*p* < 0.01 for all flavonoids in NO, *p* < 0.01 for daidzin in TNF-α; Supplementary Figures 1B,C), whereas IL-10 levels were more augmented by choerospondin and daidzin than LPS-exposed cells (*p* < 0.01 for both; Supplementary Figure 1D).

## 4 Discussion

In this study, we discovered candidate flavonoids that exert beneficial effects on NAFLD using a comprehensive strategy that combines AI-DTI and network medicine framework. The AI-DTI model provided activatory and inhibitory CTIs for all included flavonoids, and the hypergeometric test supported the reliability and accuracy of the CTI prediction results. The measured proximity successfully recovered known flavonoids that exhibited therapeutic effects on NAFLD, along with potential mechanisms. Then, we performed *in vitro* experiments using HepG2 and AML12 cells of the NAFLD model, which were triggered by FFA. The results showed that glycitin significantly attenuated lipid accumulation in hepatocytes cells, conferring its therapeutic potential against NAFLD. The potential mechanisms of glycitin were proposed and supported by molecular docking, overrepresentation analysis, and network analysis.

We believe that our study can contribute significantly to the discovery of novel candidates and clinical implications for NAFLD. To the best of our knowledge, this study is the first to systematically identify candidates for NAFLD using network proximity measure. Network-based prediction prioritizes candidate drugs under the assumption that the emergence of disease is related to the breakdown of a coordinated function of a distinct group. This hypothesis is consistent with the pathogenesis of NAFLD, which involves multifactorial pathogenic properties. Our findings also suggest that network-based approach could be a promising strategy for discovering candidate drugs for NAFLD. In addition, we used AI-DTI to predict the target of flavonoids, enabling the network-based prediction model to fully screen for potential flavonoids. Using the predicted target information, it was possible to measure the network proximity between all flavonoid targets and NAFLD-associated proteins. Notably, the proximity of glycitin, a flavonoid validated in our study, was measured solely based on predicted target information, which supports the practical usefulness of the prediction model. Considering that AI-DTI uses only 2D molecular structures of molecules for target prediction, our results suggest that combining target prediction models with network-based prediction can also be applied to discovering other natural product candidates whose target information is poorly known. We also reveal the system-level mechanism of flavonoids in NAFLD through a network-based strategy. These mechanisms can offer plausible hypotheses for beneficial effects on NAFLD of flavonoids investigated in previous clinical studies.

Experimental verification is an essential step in evaluating the accuracy and reliability of *in silico* approaches for drug discovery. Herein, we evaluated the pharmacological properties of glycitin, a soybean isoflavone, in an FFA-induced NAFLD hepatic cell model. In addition, its metabolite (glycitein) and other isoflavones (choerospondin and daidzin) were used for comparative evaluation. Consistent with our prediction, glycitin-exposed hepatic cells exhibited considerable reduction in lipid and TG accumulation, which was superior to that of other isoflavones (Figures 5A–C). In fact, there was a partial anti-obesity effect of glycitin/daidzin mixture in high-fat diet-fed mice, whose effects were shown by regulating oxidative stress (Zang et al., 2015). As corresponded to our predictions, our experimental outcome showed that glycitin has potent antioxidant property, as evidenced by ROS scavenging activity and antioxidant-related genes expression (Figures 7A–C). Indeed, anti-inflammatory activity of glycitin was demonstrated in mice models of both pneumonitis and osteoarthritis through inhibiting NF-κB pathway (Chen et al., 2019; Wang et al., 2020). These potentials would have led to the anti-NAFLD effects of glycitin *via* modulating lipid metabolic processes (Figure 7C), as we suggested using GSEA and network analysis.

Our study proposes a practical approach to identify candidate flavonoids for NAFLD. However, there are several limitations to this study with the potential for further improvement. First, the quality of the constructed compound-protein network for flavonoids is not fully guaranteed. Although we showed the reliability of our prediction results by using hypergeometric test and molecular docking, the predicted results may still contain false-positives. Also, in order to understand the predicted interaction from structurally-oriented chemistry perspective, other cheminformatics tools such as docking simulation should be employed together. Second, we considered only the native compounds in the dataset as compound lists for screening therapeutic effects, ignoring the possibility that the metabolites of the compounds were actual candidates that could exhibit actual bioactivity. Unlike small molecules, certain known therapeutic effects of natural products can be exerted by the native molecules, and by their metabolic byproducts (Demain, 2014; Koeberle and Werz, 2014; Rodrigues et al., 2016). An interesting future study would be to develop a predictive model that considers the natural product itself, as well as the interaction between the protein target and its by-products or metabolites. Finally, we selected the Phenol-Explorer database as the main source of data, more information from other databases should be considered as a future step. For example, the TCMID is by far one of the most comprehensive TCM databases (Xue et al., 2013), including 46,914 prescriptions, 8,159 herbs and 25,210 ingredients, which can greatly help to investigate more potential candidates. Despite these limitations, the proposed framework showed the potential to systematically reveal the mechanism of action underlying the beneficial effects of flavonoids on NAFLD, offering a promising strategy for mechanism-based drug development of natural products.

## Data Availability

The original contributions presented in the study are included in the article/Supplementary Material, further inquiries can be directed to the corresponding authors.
