# Supplementary figures and images for: Identifying Candidate Flavonoids for Non-Alcoholic Fatty Liver Disease by Network-Based Strategy
Source: Front Pharmacol. 2022 May 26;13:892559. doi: 10.3389/fphar.2022.892559 (PMC9204489; doi:10.3389/fphar.2022.892559)

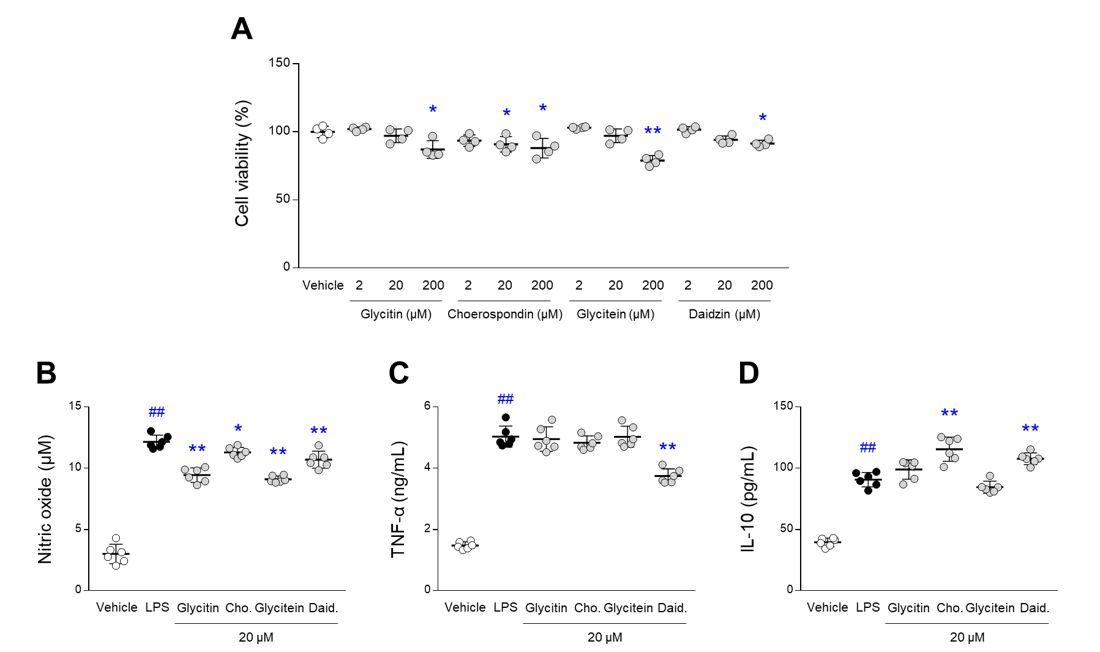

Supplement: Supplementary file 1 [file Image1.tif]
